# Vice-President Hendrick’s Disease

**Published:** 1885-12

**Authors:** 


VICE-PRESIDENT HENDRICK'S disease is said by the papers, on authority of the attending physician, to have been brain paralysis. That seems to be mere conjecture—as he was a hard mental worker—for surely, pain in the abdomen, (all he complained of, ) is not a symptom of brain paralysis; (by the bye, what *is* brain paralysis?) After a "reception," griping pain in the abdomen, attended with constipation (he was given an enema and an emetic,) would rather suggest "colic," or some derangement of digestion than a mental or nervous disease, and it seems his physician treated him for colic. But people don't usually die of colic, nor of indigestion, nor from overloaded stomach, especially after an emetic and an enema. The diagnosis of "brain paralysis" wont do.

